# Biventricular ICD Placement Percutaneously Via the Iliac Vein: Case Reports and a Review

**DOI:** 10.19102/icrm.2017.080702

**Published:** 2017-07-15

**Authors:** Steven L. Higgins

**Affiliations:** ^1^Department of Cardiology, Scripps Memorial Hospital, La Jolla, CA

**Keywords:** Abdominal, biventricular implantable cardioverter-defibrillator, cardiac resynchronization therapy, femoral vein, iliac vein

## Abstract

Cardiac resynchronization therapy (CRT) has been demonstrated to improve symptoms of heart failure. As a result, it has become the standard of care in selected patients, and is commonly completed with three leads placed via an upper-extremity vein. However, in rare situations, such as in the case of superior vena cava occlusion, venous access is not possible via the upper extremity. It is in such instances that alternative means must be sought. Here, two patients who received a CRT defibrillator via an iliac vein approach with a mid-abdominal generator are introduced, and a review of the techniques used is presented. Technical aspects to this approach are discussed, including iliac venous access, defibrillation electrode positioning, coronary sinus access, and lead tunneling to an abdominal generator for patient comfort. This approach should be considered when vascular access is compromised, at least until combined leadless CRT pacing and subcutaneous implantable cardioverter-defibrillator devices become available and feasible for use.

## Introduction

Since 1980, isolated case reports have described cardiac implantable electrical devices (CIEDs) inserted via lower-extremity access.^1^ Here, we present two cases and review an approach designed to minimize lead and device complications utilizing iliac vein access, tunneling to a mid-abdominal generator location. We also provide recommendations regarding access and management.

### Case 1

**Figure 1:**
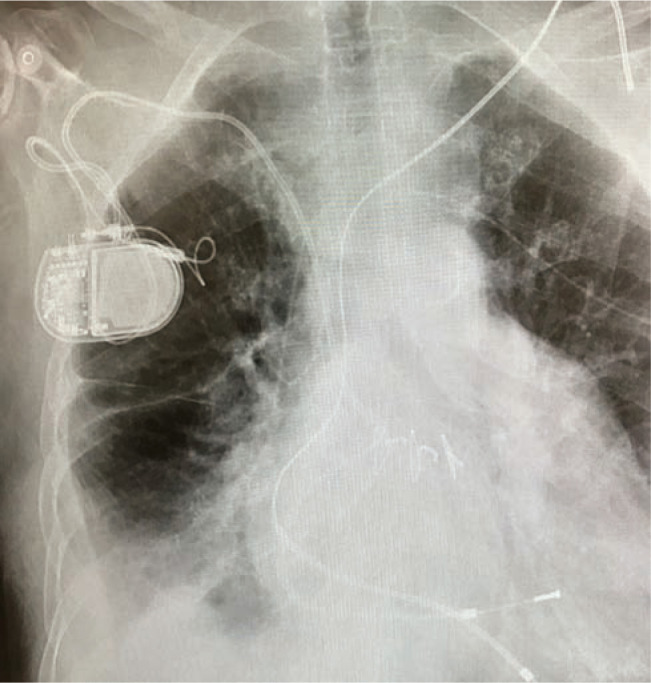
Preoperative chest X-ray reveals only two transve-nous electrodes despite 40 years of pacing therapy. Also noteworthy for marked cardiomegaly and a horizontal substernal approach.

An 80-year-old female presented to the clinic for pacemaker upgrade. In 1963, she had undergone surgical repair of an atrial septal defect by Dr. Michael DeBakey in Houston, TX. In 1977, she developed complete heart block, complicating her permanent atrial fibrillation (AF). Since then, she has had eight permanent pacemaker insertions, all of which have been single-chamber ventricular devices. In 2017, the patient was referred with pacemaker battery depletion but also with the presentation of a new non-ischemic cardiomyopathy and an ejection fraction (EF) of 25% with normal coronary arteries noted by angiogram. A chest X-ray revealed only two intracardiac leads **(Figure 1)**.

**Figure 2:**
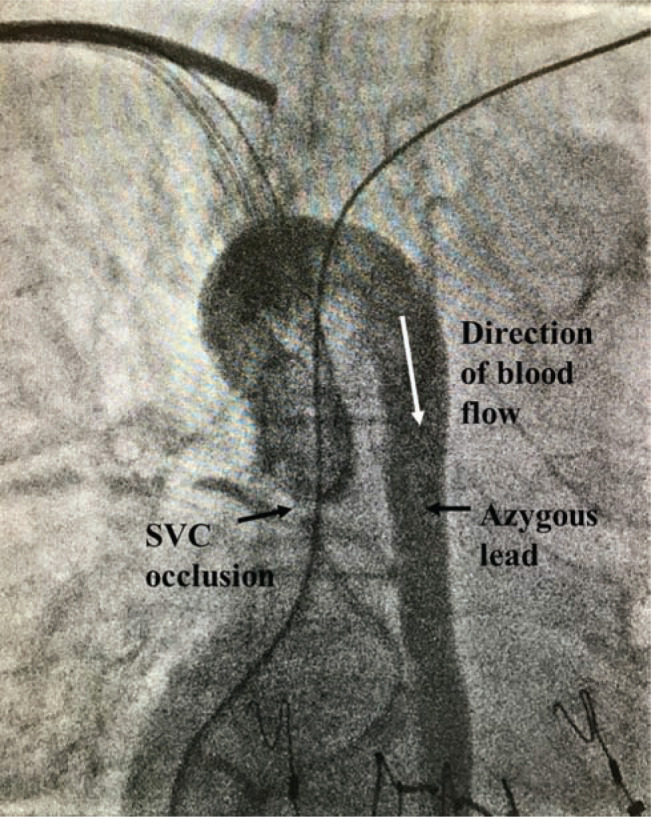
Intraoperative venogram from a right subclavian sheath shows total occlusion of the SVC with retrograde flow via the azygous vein to the inferior vena cava.

**Figure 3:**
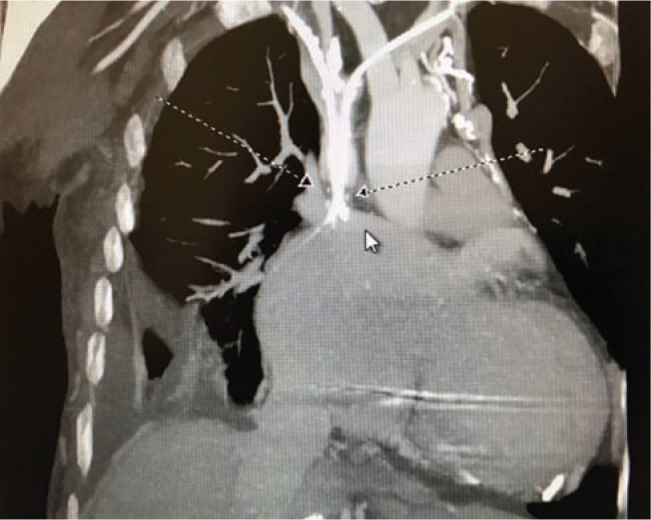
Postoperative computed tomography angiogram shows complete occlusion of the superior vena cava just above the right atrium (arrows).

Plans were made for this patient to receive an upgrade to a biventricular implantable cardioverter-defibrillator (ICD). However, at the time of surgery, guidewires could not be passed into the right heart either via the right or left subclavian approach because of complete occlusion of the superior vena cava (SVC). An angiogram **(Figure 2)** showed the presence of a large azygous vein connecting the SVC retrograde with the inferior vena cava. Therefore, only the pacemaker generator was changed. Subsequently, a computed tomography angiogram was performed, and confirmed the complete SVC occlusion **(Figure 3)**.

Despite ongoing medication therapy, the patient’s symptoms of advanced heart failure (HF) persisted, and she requested further assistance. Cardiac surgical consultation suggested that the risks of placing an epicardial left ventricle (LV) lead were excessive.

**Figure 4:**
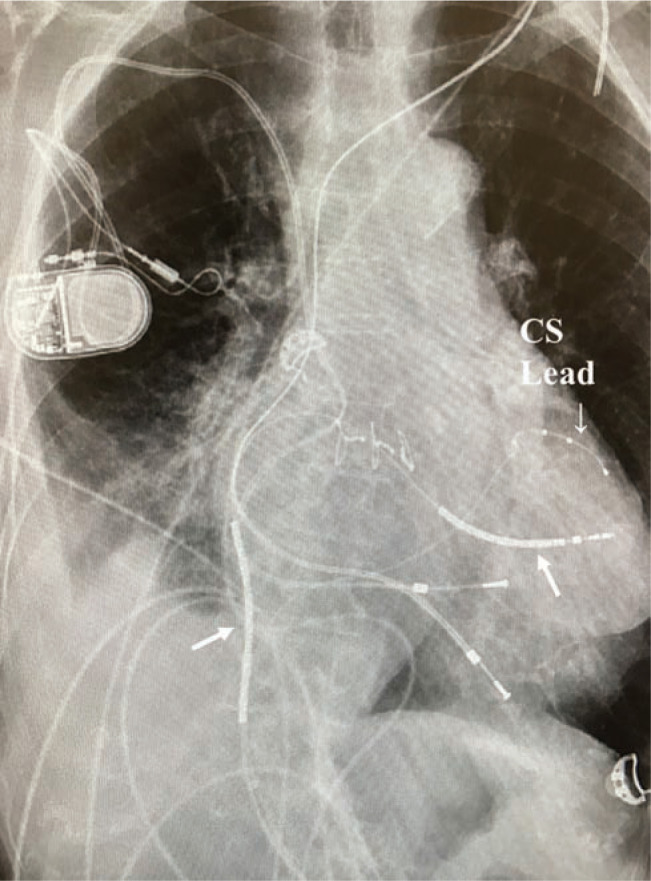
Postoperative posterioranterior chest radiograph showing two new transvenous leads inserted via the iliac vein. The CS lead is labeled, present in a basal posterolateral location. Note the insertion of the RV shocking lead in the mid-RV septum, chosen to separate the two defibrillation coils with RV myocardium between them (arrows).

A transvenous femoral approach was utilized. Venous access was first obtained via the right femoral vein using a guidewire and a roadmap iliac angiogram. Next, a percutaneous approach was utilized above the inguinal ligament to access the external iliac vein, above the stress point of the inguinal crease. Two separate iliac veni-punctures were used for placing a right ventricular (RV) dual-coil shocking electrode, placed in the mid-RV septum with additional slack provided for optimization of the vector between the two shocking coils. Successful conversion of induced ventricular fibrillation (VF) was confirmed. Using a right-sided curved guiding sheath, a coronary sinus quadripolar pacing electrode was placed without difficulty in a basal posterolateral LV location **(Figure 4)**. The dual-coil defibrillation electrode was placed with its tip on the mild RV septum and the proximal coil advanced to near the tricuspid valve to maximize the amount of myocardial mass between the two electrodes (arrows, **Figure 4)**. Owing to the patient’s permanent AF, even after cardioversion, no atrial lead was placed.

**Figure 5:**
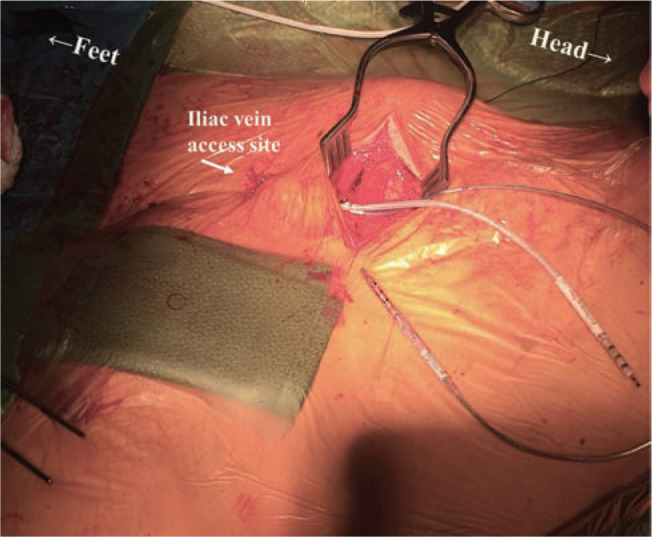
Intraoperative photograph, oriented with the patient’s head to the right. The leads were placed via iliac venous access and tunneled to a mid-abdominal wall subcutaneous pocket.

**Figure 6:**
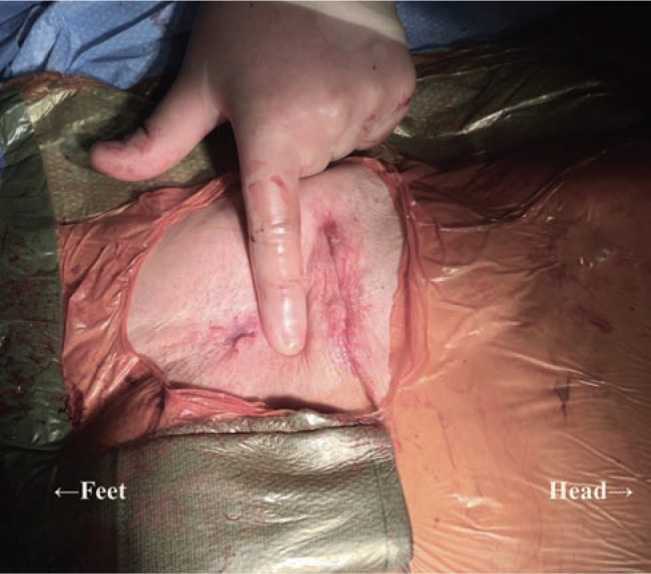
Immediate postoperative view, with similar orientation, showing the two incisions required for venous access and the device pocket.

To minimize discomfort of the device near the inguinal region, a pocket was created superiorly in the mid-right abdominal wall. The leads were tunneled from the iliac entry site to the pocket and attached to the new device (**Figures 5 and 6)**. Induced VF was terminated with a 16-joule (J) shock using a dual-coil vector excluding an active can.

Five months postoperation, the patient displayed dramatic improvement in her HF symptoms, presumably from cardiac resynchronization therapy (CRT). At this time, her New York Heart Association HF class improved from IV to II.

### Case 2

A 74-year-old male received a left-sided dual-chamber ICD in 2007 for an EF of 20% from an ischemic cardiomyopathy. Following the initial implantation, he required reoperation for a lead fracture. Subsequently, he developed a device infection and bacteremia with methicillin-resistant *Staphylococcus aureus* (MRSA). The entire ICD system was explanted using a laser lead system. The infection healed and a new device was subsequently placed on the contralateral side.

Two years later, the patient developed another MRSA infection involving the now right-sided biventricular ICD system. Again, the system was explanted and the infection was treated. Angiograms documented bilateral occlusion of the subclavian and axillary venous systems. Cardiac surgical consultation was sought regarding placement of epicardial leads for a biventricular ICD. However, because of the patient’s comorbidities, the surgical risk was considered excessive.

**Figure 7:**
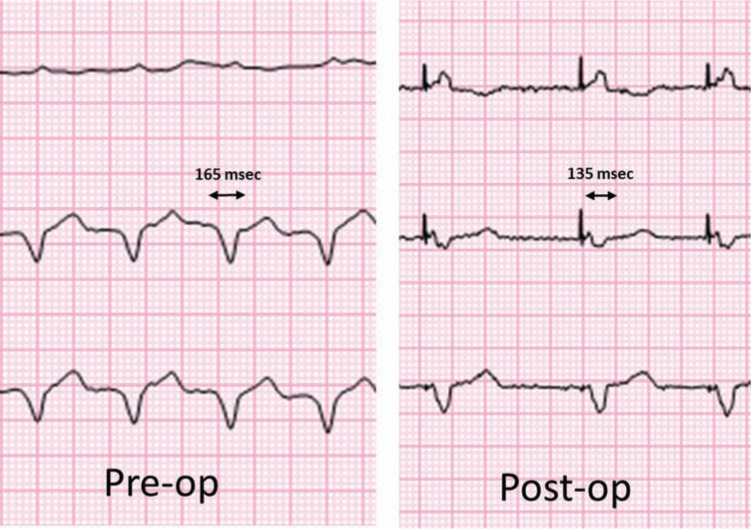
A three-channel electrocardiogram showing a decrease in QRS duration with the resumption of biventricular pacing. Using 12-lead analysis, the QRS duration decreased from 165 to 135 ms with CRT pacing.

**Figure 8:**
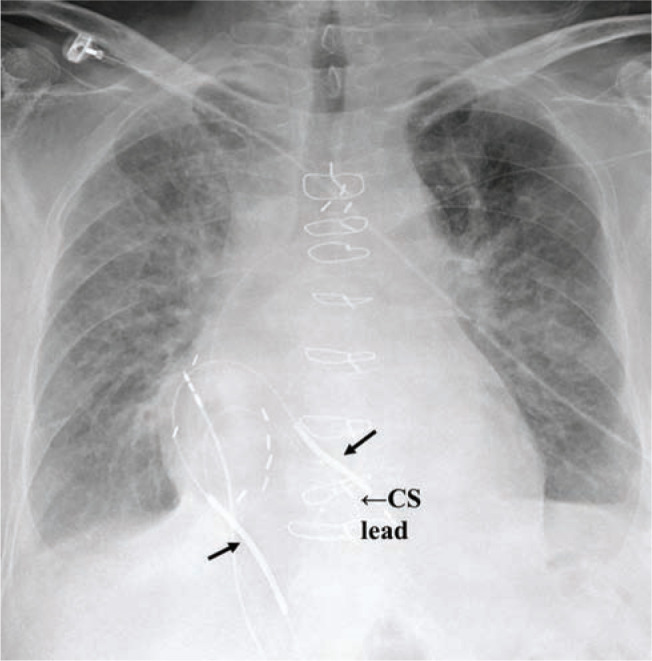
Postoperative AP chest radiography for Case 2 showing three intracardiac leads. The right atrial lead is along the lateral mid-RA, the LV CS lead is a proximal middle cardiac vein (labeled), and the RV lead in the lower septum with separation between the defibrillation coils (black arrows).

At this point, symptoms of progressive HF and symptomatic sinus bradycardia developed, and the patient’s EF diminished to less than 15%. A new biventricular ICD was placed via the right iliac vein using a technique similar to that described above in Case 1. Three leads were inserted in the iliac vein, with a right atrial lead placed in the lateral high right atrium, requiring a lead extension adapter. The LV lead was placed in a basal middle cardiac vein. Coronary sinus (CS) access required the use of a steerable quadripolar electrophysiology (EP) catheter, with advancement of the CS guiding sheath over it. Despite a medial and basal LV CS pacing site (near the CS os), the QRS duration narrowed with LV pacing **(Figure 7)**. The RV dual-coil defibrillation lead was placed in the lower-RV septum with additional right atrial slack enabled to allow for the proximal coil to reside within the heart **(Figure 8)**. Induced VF was terminated with a 16-J shock using a dual-coil vector, excluding an active can. Testing using a single-coil vector or active can was not performed.

Three months after surgery, the patient had improved clinically, rescinding his request for a do-not-resuscitate order. His EF increased from 15% to between 25% and 30%.

## Discussion

The benefits of CRT were first demonstrated with an epicardial approach, followed by the standard transvenous approach typically used today.^1^ Dating back to 1980, isolated case reports have demonstrated the capability of a transvenous approach via the femoral vein to be used for CIED insertion.^2^ In this case series, we incorporated an adaptation of the femoral approach for biventricular device insertion, utilizing the iliac vein with leads tunneled to a mid-abdominal device pocket. Techniques and advances from the initial femoral approach include:

- percutaneous iliac vein access to minimize stress lead fracture associated with femoral vein insertion below the inguinal crease;
- dual-coil defibrillation electrode insertion with care taken to optimize defibrillation vector;
- CS access utilizing techniques from the EP laboratory;
- the availability of lead extensions; and
- abdominal wall generator with lead tunneling for patient comfort.

Although most prior case reports utilize femoral venous access, the iliac approach was first described in 2010.^3^ We and others now utilize the iliac vein approach due to the increased risk of lead fracture and thrombophlebitis described with femoral venous device insertions.^4^ However, with this approach, the risk for a retroperitoneal hematoma is also increased and is more commonly observed with femoral angiography. Iatrogenic retroperitoneal hematoma occurs in from 0.15% to 0.5% of routine angiograms via the femoral artery.^5,6^ The risk is higher in those with arterial punctures above the inguinal ligament.^7^ We presume that supra-inguinal venous access also increases this risk.

**Figure 9:**
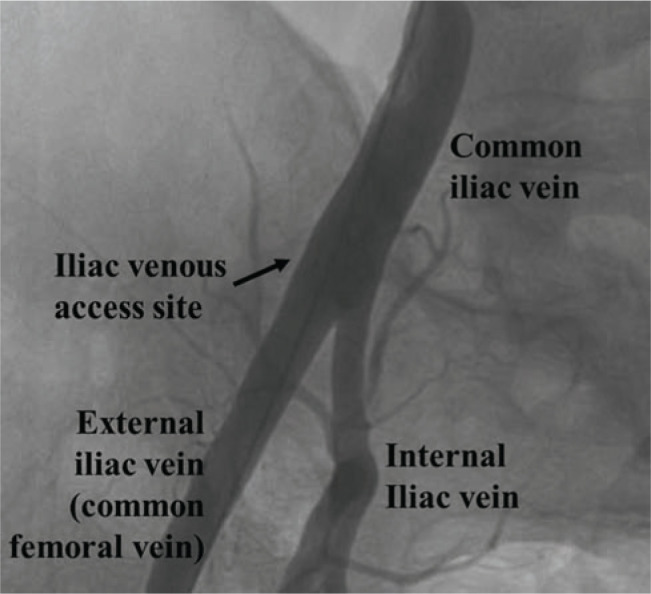
A right femoral venogram showing the external and internal iliac veins forming the common iliac vein near the pelvic crest. Inferiorly, the external iliac vein is called the common femoral vein. It is formed by the combination of the superficial femoral and the greater saphenous veins (not shown).

In our cases, we performed a roadmap femoral venous angiogram to help with percutaneous access to the external iliac vein **(Figure 9)**. Others have utilized ultrasound localization for similar access with the goal of accessing the iliac vein prior to its posterior angulation above the pelvic crest. As opposed to direct percutaneous retroperitoneal venous access, access achieved at a point just above the inguinal ligament presumably decreases the risk of retroperitoneal bleed.^8^ However, despite its more superior location, the pelvic region is not a desirable location for device insertion still necessitating lead tunneling.

Coronary sinus access from the femoral vein is performed routinely in the EP laboratory for temporary catheter insertion. However, the CS delivery sheaths provided with CRT systems are predominantly designed for upper-extremity access. In one of our two cases, we required a steerable quadripolar EP catheter for access to the CS, as the guiding sheath (for transvenous lead delivery) alone could not be positioned in the CS. The CS guiding sheath was then advanced over the EP catheter, the catheter was removed, and the CS lead was placed in a fashion similar to that for upper-extremity access. Subsequently, utilizing a cutting tool, the guiding sheath was removed.

Another challenge to successful abdominal device placement is lead length. These leads have to traverse from the mid-abdominal generator inferiorly to the inguinal vein access point, and then superiorly back to the heart. Fortunately, ICD and CS leads are currently available in lengths of 85 cm to 100 cm, though atrial pacing leads tend to be shorter. In the one case that utilized a 60-cm atrial lead, the length was inadequate so a 10-cm lead adapter was added (model BIV79714; Oscor Inc., Palm Harbor, FL, USA). In our case, adequate slack was present, but if this had not been so, a second lead adapter could have been incorporated. Lead dislodgement of femoral leads, particularly of those in the right atrium, has been reported to be as high at 21%.^9^ These authors suggest that improved lead length, or the use of an extension as described, may minimize that risk.

Skin erosion has been described to occur in cases with devices placed in the lower abdominal wall near the inguinal crease.^7^ However, historical experience with tunneled leads directed to an anterior abdominal wall location have shown this to be safe and efficacious.^10^ Similar to pectoral placement, options exist for subcutaneous and submuscular approaches in the abdominal wall. For most adults, currently available smaller ICD generators have a size adequate for subcutaneous abdominal wall placement as opposed to the need for the placement of older, large devices under the rectus sheath.^11^ Despite today’s smaller device volumes, a small adult or child could still benefit from a subrectus approach.^12,13^

Historically, transvenous ICD leads were first inserted via an upper-extremity vein and tunneled to the abdomen. The abdominal location was required due to the excessive volume and weight of early ICDs, which were nearly 300 grams (g), as compared with those weighing under 100 g today.^14^ As device size diminished, pectoral implantation became routine.

Today, with concerns about the future risks associated with the extraction of dual-coil leads, single-coil leads are increasing in popularity.^15^ However, we were concerned that the use of a single-coil lead using a remote abdominal generator as an active can may not provide adequate safety for successful defibrillation. One earlier case report from our institution did describe a femoral placement that required the intraoperative addition of a subcutaneous array electrode for adequate defibrillation safety.^16^ Therefore, for the cases described, we elected to place a dual-coil lead and provided additional slack, so that the proximal electrode resided in the low right atrium near the tricuspid valve. In addition, we placed the RV lead above the RV apex to increase the myocardial mass between the two coils. The generator in this case was made inactive for defibrillation, commonly called a cold can approach. Both patients underwent successful defibrillation testing with a delivered 16-J shock, providing an acceptable safety margin for the 40-J implanted defibrillator.

A subcutaneous ICD is currently available commercially, and is useful for patients with venous access challenges.^17^ Unfortunately, CRT pacing is not currently available with this system. In both of our patients, severe symptoms from HF warranted the transvenous CRT system as described.

When upper-extremity venous access is not possible, epicardial lead access via thoracotomy remains an option. However, both of these patients had prior thoracotomies and a severe cardiomyopathy, resulting in an estimated high surgical risk for repeat thoracotomy, even with consideration for a video-assisted thorascopic approach. Recovery from this transvenous procedure was more rapid than it would have been after thoracotomy.

Recently, advances have been made with leadless pacing technologies, routinely inserted via temporary femoral venous sheaths.^18^ Future advances may include wireless dual-chamber pacing systems (with integrated wireless pacing). Subsequently, leadless LV pacemakers may provide resynchronization therapy without the need for intracardiac leads.^19^ Potentially, these systems could be coupled with a subcutaneous ICD.^20^ In the not-too-distant future, patients with upper-extremity venous access challenges may be best served with leadless pacing and subcutaneous defibrillation options.

## Conclusions

The benefits of CRT have become widely recognized in the management of advanced HF. When access from an upper-extremity vein is not possible, the implantation of a biventricular ICD system from an iliac vein approach is feasible, though there are several technical challenges. Care must be taken to minimize vascular complications such as thrombophlebitis and retroperitoneal hemorrhage. Lead length limitations may necessitate the use of a lead extension. Coronary sinus lead placement may require specialized techniques adapted from the EP laboratory. To optimize defibrillation efficacy, we encourage the use of a dual-coil defibrillation lead with positioning of the proximal coil near the tricuspid valve and elimination of the active can. Lead tunneling from the iliac access point to a mid-abdominal location provides improved postoperative patient comfort. In the future, advances in leadless technology may entirely eliminate the need for transvenous leads, even for cardiac resynchronization with defibrillation capability.

## Acknowledgments

The author wishes to thank Amy Cardella and Robin Jass of Biotronik, Inc., and Ds. Eric Hong and George Wesbey for assistance with the procedures discussed in this manuscript.

## References

[1] Higgins SL, Hummel JD, Niazi IK (2003). Cardiac resynchronization therapy for the treatment of heart failure in patients with intraventricular conduction delay and malignant ventricular tachyarrhythmias. J Am Coll Cardiol..

[2] Ellestad MH, Caso R, Greenburg PS (1980). Permanent pacemaker implantation using the femoral vein: A preliminary report. Pacing Clin Electrophysiolol..

[3] Gopinathannair R, Campbell DN, Goldsmith G, Olshansky B (2010). Transiliac biventricular ICD implantation: the next step in patients with upper extremity venous occlusion. J Innov Cardiac Rhythm Manage.

[4] Trigano A, Paganelli RF, Alimi Y, Juhn C (1997). Surgical interruption of the left inferior vena cava following the transfemoral implantation of a permanent pacing lead. Pacing Clin Electrophysiolol..

[5] Levine GN, Kern MJ, Berger PB (2003). Management of patients undergoing percutaneous coronary revascularization. Ann Intern Med..

[6] Lubavin BV (2004). Retroperitoneal hematoma as a complication of coronary angiography and stenting. Am J Emerg Med..

[7] Chan YC, Morales JP, Reidy JF, Yalor PR (2008). Management of spontaneous and iatrogenic retroperitoneal haemorrhage: conservative management, endovascular intervention or open surgery?. Int J Clin Pract..

[8] Jiang J, Ding X, Zhang G, Su Q, Wang Z, Hus S (2010). Spontaneous retroperitoneal hematoma associated with iliac vein rupture. J Vasc Surg..

[9] Mathur G, Stables RH, Heven D, Ingram A, Sutton R (2001). Permanent pacemaker implantation via the femoral vein: An alternative in cases with contraindications to the pectoral approach. Europace..

[10] Higgins SL, Abdominal generator techniques (1997). The Implantable Cardioverter Defibrillator. A videotape and manual..

[11] Higgins SL (1998). Implantation techniques. Card Electrophysiol Rev..

[12] Lichtenstein BJ, Bichell DP, Connolly DM, Lamberti JJ, Shepard SM, Seslar SP (2010). Surgical approaches to epicardial pacemaker placement: does pocket location affect lead survival?. Pediatr Cardiol..

[13] Sabti HA, Menon RG, Maddali MM, Valliattu J (2008). Wandering permanent pacemaker generators in children: a case series. J Med Case Rep..

[14] Higgins SL (1997). The Implantable Cardioverter Defibrillator. A videotape and manual.

[15] Larsen JM, Hjortshoj SP, Nielsen JC (2016). Single-coil and dual-coil defibrillator leads and association with clinical outcomes in a complete Danish nationwide ICD cohort. Heart Rhythm..

[16] Perzanowski C, Timothy P, McAfee M, McDaniel M, Meyer D, Torres V (2004). Implantation of implantable cardioverter-defibrillators from an ileofemoral approach. J Interv Card Electrophysiol..

[17] De Maria E, Olaru A, Cappelli S (2015). The entirely subcutaneous defibrillator (S-ICD): state of the art and selection of the ideal candidate. Curr Cardiol Rev..

[18] Higgins S, Rogers JD (2014). Advances in pacing therapy: examining the potential impact of leadless pacing therapy. J Innov Cardiac Rhythm Manage..

[19] Mullens W, Nijst P (2017). Leadless left ventricular pacing: another step toward improved CRT response. J Am Coll Cardiol..

[20] Rutzen-Lopez H, Silva J, Helm RH (2016). Leadless cardiac devices-pacemakers and implantable cardioverter defibrillators. Curr Treat Options Cardiovasc Med..

